# Stray Magnetic Field Variations and Micromagnetic Simulations: Models for Ni_0.8_Fe_0.2_ Disks Used for Microparticle Trapping

**DOI:** 10.3390/mi15050567

**Published:** 2024-04-26

**Authors:** Gregory B. Vieira, Eliza Howard, Prannoy Lankapalli, Iesha Phillips, Keith Hoffmeister, Jackson Holley

**Affiliations:** Department of Physics, Rhodes College, Memphis, TN 38112, USA

**Keywords:** patterned magnetic thin films, magnetic particle transport, magnetic fields

## Abstract

Patterned micro-scale thin-film magnetic structures, in conjunction with weak (~few tens of Oe) applied magnetic fields, can create energy landscapes capable of trapping and transporting fluid-borne magnetic microparticles. These energy landscapes arise from magnetic field magnitude variations that arise in the vicinity of the magnetic structures. In this study, we examine means of calculating magnetic fields in the local vicinity of permalloy (Ni_0.8_Fe_0.2_) microdisks in weak (~tens of Oe) external magnetic fields. To do this, we employ micromagnetic simulations and the resulting calculations of fields. Because field calculation from micromagnetic simulations is computationally time-intensive, we discuss a method for fitting simulated results to improve calculation speed. Resulting stray fields vary dramatically based on variations in micromagnetic simulations—vortex vs. non-vortex micromagnetic results—which can each appear despite identical simulation final conditions, resulting in field strengths that differ by about a factor of two.

## 1. Introduction

Micromagnetic particles yield a tool in biology for the isolation and quantification of biomolecules such as DNA [1,2], RNA [3], and proteins [4], as well as whole cells from a heterogeneous mixture [5]. Upon conjugation of a magnetic microparticle or particles to a target entity, applications of magnetic fields and field gradients are used to isolate a target from a mixture for the purpose of detecting disease, for example by isolating circulating tumor cells [6], RNA from H1N1 viruses [7], and RNA from SARS-CoV-2 [8], including in wastewater samples [9,10]. Furthermore, applying magnetic fields to particles in a fluid environment can be used to treat wastewater [11], to modify physical characteristics of particle populations such as inter-particle spacing [12], as well as to guide fluids in a lab-on-chip environment [13]. Also of interest is targeted drug delivery at specified locations in the body while minimizing unwanted exposure, done by employing magnetic implants to concentrate magnetic nanoparticles and deliver drugs at desired locations [14,15,16]. Additionally, fluid-borne magnetic disks have shown promise as a means of targeting cancer cells in the body via localized heating (i.e., magnetic hyperthermia), delivering antitumor drugs, or mechanical disruption of cancer cells (i.e., by applying forces and torques) [17]. The above uses of magnetic particles involve manipulation via magnetic interactions, necessitating a thorough understanding of magnetic fields and the field gradients that are the cause of these forces.

Traditionally, the manipulation necessary for isolating or applying forces to magnetic microparticles is performed using handheld and otherwise macroscopic magnets, but it has been shown that interactions between microscopic thin-film features in magnetic materials are sufficient to capture fluid-borne particles, using both discrete [18,19] and continuous (i.e., wire) [20,21,22] patterned elements. These trapping and transport mechanisms are advantageous because of their precision, as trap sizes are governed by the geometry of the patterned materials, as well as their scalability, as modern lithography techniques allow for many traps to be engineered on a surface.

By applying and varying magnetic fields, the strength of magnetic forces can be tuned carefully, allowing for the manipulation of magnetic particles along pre-programmed routes on a surface [23,24,25,26,27,28], the tuning of trap strengths by external fields [18], the tuning of particle velocities by applying external fields [29], and the varying of Brownian fluctuations by small changes in external field strengths [30]. As small changes in fields can lead to large ramifications in particle behavior, to understand and successfully predict particle motion, having robust mathematical models of magnetic fields from magnetic thin-film devices is essential.

Analytical models exist and are well-understood for disks uniformly magnetized in a direction perpendicular to the top/bottom face [31,32]; however, for the patterned disks discussed in this paper, the width-to-thickness aspect ratio is sufficiently large to restrict magnetization to the plane of the film. Additionally, for in-plane magnetized disks, semi-analytical techniques are employed to calculate magnetic fields when the magnetization is uniform [33,34]. However, at low externally applied fields, disk magnetization is far from uniform, so other methods are required for calculating disk fields.

In this study, we investigate the local magnetic fields produced by permalloy (Ni_0.8_Fe_0.2_) magnetic disks. In particular, as it has been shown that these disks are capable of microparticle transport at low external fields—less than 100 Oe—we investigate magnetic field profiles from disks in the presence of low applied fields and as such, we require the use of a realistic magnetization landscape in our models. We employ simulations based on the Object Orientated Micromagnetic Framework (OOMMF) [35] to calculate local stray fields from the magnetic disks. While micromagnetic simulations such as OOMMF are commonly used to describe particles trapped and transported by thin-film structures [27,28,29,36,37,38,39], we focus on the variations that arise from repeated OOMMF simulation runs and notice differences that arise in resulting disk fields. Additionally, as methods based on the OOMMF rely on 2D summation over a discretely defined magnetization landscape for an individual field calculation and are, hence, computationally slow, we discuss a fitting method for faster computation of fields. 

## 2. Materials and Methods

### 2.1. Particle Trapping and Transport and Effects of Externally Applied Magnetic Fields

**Figure 1:**
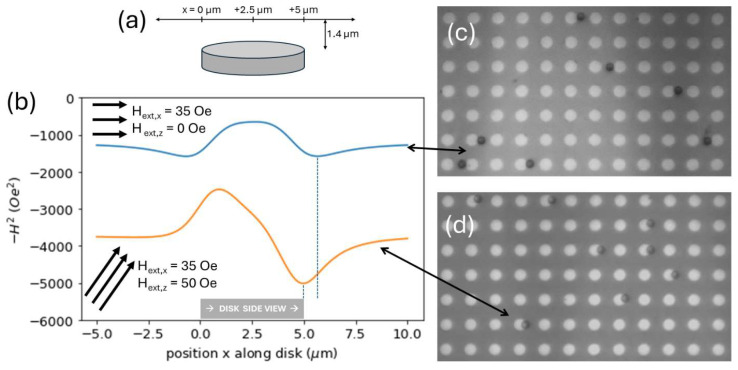
(**a**) Cartoon showing location, indicated by number line, where magnetic fields are calculated in the vicinity of a magnetic disk. (**b**) To show how the potential energy varies in the vicinity of a magnetized disk, we plot *−H*^2^ (proportional to potential energy for a linearly magnetizable magnetic bead) vs. position at a height of 1.4 μm above the disk. We plot *−H*^2^ magnetized by and in the presence of an in-plane external magnetic field of magnitude 35 oersted (blue) and a magnetic field with a horizontal and vertical component of 35 oersted and 50 oersted, respectively (orange). In the absence of the vertical component of the magnetic field, the potential energy curve ($-H^{2}$) appears symmetrical, with traps (potential energy minima) on either side. Upon application of a 50 Oe out-of-plane field in addition to the in-plane field, the potential energy curve is no longer symmetrical, as the trap strengthens on one side of the disk while becoming repulsive on the other. Note that the potential energy is much lower (more negative) in the case of the multiple-component field because the total field magnitude is larger. (**c**) Magnetic beads on either side of disks while in the presence of an in-plane, 35 Oe field, corresponding with the blue potential energy curve. (**d**) Magnetic beads exclusively on the right side of disks because of the addition of a 50 Oe out-of-plane field, corresponding with the orange potential energy curve. We note that beads in (**c**) overlap the disks less than in (**d**), consistent with the locations of the energy minima in (**b**) (see dotted lines).

Upon magnetizing disks by the application of a magnetic field in an in-plane direction (i.e., to the right in Figure 1a), traps are formed at opposite ends of magnetic disks—magnetizable particles such as superparamagnetic microparticles, or “beads”, are capable of being trapped at one of two sides of the disk (i.e., the north or south pole), as seen in Figure 1c. Upon the application of an out-of-plane magnetic field $H_{ext,z}$, preference is given to one pole, as seen in Figure 1d, while the other is repulsive. To illustrate this, we consider a superparamagnetic bead that is linearly magnetizable by an external field such that its magnetic moment ${\vec{m}}_{bead}=\chi V\vec{H}$, where χ is the particle’s magnetic susceptibility, V is the particle’s volume, and $\vec{H}$ is the net magnetic field the particle is in. The resulting force on the bead from a spatially varying magnetic field is [19]

(1) $$\vec{F}=\frac{1}{2}\mu_{0}\chi V\vec{\nabla }H^{2}$$

and since generally force $\vec{F}=-\vec{\nabla }U$, the resulting potential energy U, then, is given by the equation below.

(2) $$U=-\frac{1}{2}\mu_{0}\chi VH^{2}$$

We note that the potential energy is proportional to the negative square of the field $\vec{H}$ where $\vec{H}$ is defined as the net magnetic field from all sources—in this study, the sum of fields from disks (${\vec{H}}_{disk}$) and externally applied fields (${\vec{H}}_{ext}$). In Figure 1b we plot $-H^{2}$ vs. position along a line parallel to the disk diameter at a height of 1.4 µm, the radius of a commonly used magnetic bead, above the disk center. For a magnetic disk in the presence of an externally applied 35 Oe, rightward facing field, the plot shows two energy minima—one on each edge of the disk, shown in blue in Figure 1b. For a disk in the presence of a 35 Oe in-plane and 50 Oe out-of-plane field, one energy minimum is reduced, suggesting a stronger trap at the rightmost disk edge, and the other energy minimum is inverted, suggesting a repulsive disk edge. This effect is reversed if $H_{ext,z}$ is down instead of up.

For this study, we exclusively focus on permalloy disks of a particular size: 5 μm diameter, 40 nm thickness. As permalloy is a soft magnetic material, the application of a magnetic field is required to produce magnetic traps—when fields are turned off, trapped particles are released from traps on disk peripheries and are observed to be carried away from disks by Brownian fluctuations or even gentle fluid flow.

While the externally applied magnetic fields are easy to measure, it is difficult to measure small variations in fields near the surface due to the patterned entities. This study focuses on methods for calculating these local “stray” magnetic fields from these disks (${\vec{H}}_{disk}$). We also explore variations that arise due to differences in micromagnetic simulations that guide the field calculations. 

### 2.2. Methods for Field Calculations

**Figure 2:**
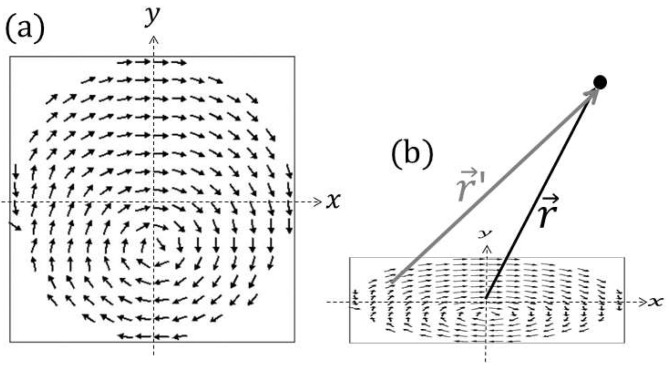
(**a**) OOMMF simulation of the magnetization landscape of a 5 µm diameter, 40 nm thick permalloy disk in a magnetic field of components $H_{ext,x}=$ 35 Oe and $H_{ext,z}=$ 50 Oe. (**b**) Coordinate system and definitions of position vectors $\vec{r}$ and ${\vec{r}}^{\prime }$ used for calculating magnetic fields resulting from this disk configuration.

Magnetic fields from disks were calculated first by simulating micromagnetic details using the OOMMF, then by calculating fields a defined point away from the disk either by summing dipole fields or summing Coulomb fields from a bound magnetic charge density distribution. As an example of an OOMMF simulation used, Figure 2a shows a 5 μm diameter, 40 nm thick permalloy disk in an external field configuration ($H_{ext,x}=$ 35 Oe, $H_{ext,z}=$ 50 Oe) used for particle manipulation [40]. The figure shows a representation of the varying magnetization directions of portions, or “cells”, of the disk generated by the OOMMF simulation. We refer to this spatially varying disk magnetization as a “magnetization landscape”, and it is indicated by the arrows in Figure 2a. In this case, the magnetization landscape appears as a vortex pattern. In the figure, the x- and y-axes are defined. (The z-axis is normal to the disk plane). We place the disk such that the center of the bottom of the disk is at the origin of our coordinate system, and we define a vector $\vec{r}$ that describes the location of the field being calculated. Furthermore, we define a vector, ${\vec{r}}^{\prime }$, from a cell of the micromagnetic simulation to the point in space defined by $\vec{r}$. This vector ${\vec{r}}^{\prime }$ is varied as we sum over these cells when calculating fields.

We used the default parameters from the OOMMF for permalloy for our simulation: saturation magnetization $M_{S}$ = 8 × 10^5^ amps/meter, the exchange constant of A = 13 × 10^−12^ joules/meter, a damping coefficient of 0.5, and no effect from crystalline anisotropy (i.e., K1 = 0 joules/meter^3^). The simulation uses a cell size of 40 nm × 40 nm × 40 nm (arrows in the figure are averaged over several cells for display purposes) and each dipole has a vector magnetic moment of $\vec{m}$, where the moment’s magnitude is constant (and equals $M_{S}V$, where $M_{S}$ is permalloy’s saturation magnetization and V is the volume of the cell, in this case, (40 nm) × (40 nm) × (40 nm)). 

By summing the field contribution of each dipole, the stray field ${\vec{H}}_{disk}$ at position $\vec{r}$ can be calculated by the equation for a field from a dipole:

(3) $${\vec{H}}_{disk}\left(\vec{r}\right)=\frac{1}{4\pi }\sum_{\begin{array}{c} all\\ cells \end{array}}\left[\frac{3\left({\vec{m}}_{i}\cdot {\vec{r}}^{\prime }\right){\vec{r}}^{\prime }}{{\left|{\vec{r}}^{\prime }\right|}^{5}}-\frac{{\vec{m}}_{i}}{{\left|{\vec{r}}^{\prime }\right|}^{3}}\right]$$

where ${\hat{r}}^{\prime }$ is the vector from a cell to the point at which the field is calculated, as seen in Figure 2b, and ${\vec{m}}_{i}$ is the moment of the *i*th cell.

Similarly, the field can be calculated by considering a bound charge density, $\rho_{m}$, that arises when the magnetization ($\vec{M}$) has a nonzero divergence:

(4) $$\rho_{m}=-\vec{\nabla }\cdot \vec{M}=-\frac{1}{V}\left(\vec{\nabla }\cdot \vec{m}\right)$$

noting that magnetization is related to the magnetic dipoles by $\vec{M}=\vec{m}/V$. This divergence in the magnetic moment is approximated using nearest neighbor cells. 

**Figure 3:**
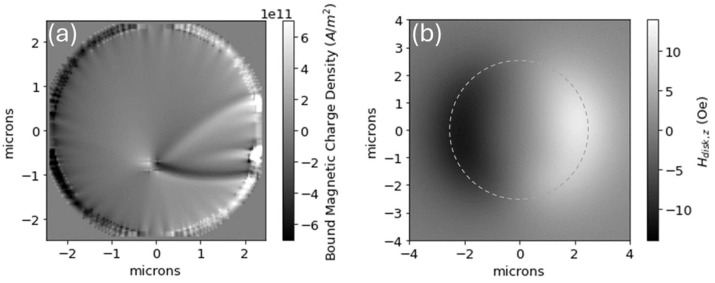
(**a**) Map of bound magnetic charge density for the magnetization landscape shown in Figure 2, defined as the negative of the divergence of the disk’s magnetization. The bound charge is mostly found near the disk periphery. (**b**) The z-component of the magnetic field 1.4 um above the disk, calculated from the charge density in (**a**), showing that the vortex structure is not apparent in the resulting magnetic field landscape. The dashed circle indicates the disk periphery (as the image length scale is different in (**a**) vs. (**b**)).

Figure 3a shows the whereabouts of the bound magnetic charge density used for field calculations. What results is a charge distribution that resides primarily near the disk periphery—white (positive bound charge density) on the right side of the disk, black (negative bound charge density) on the left, and gray (comparably little bound charge density) throughout most of the disk. While most bound charge density resides on the periphery, some bound charge density resides in the vicinity of the vortex center and stretches toward the periphery. These deviations caused by the vortex structure—that not all charge is on the periphery as it would be for a fully magnetized disk—are blurred when examining the resulting magnetic field 1.4 microns above the disk, as seen in Figure 3b. 

Noting that the charge in each cell (indexed as i) is calculated as $q_{i}=\rho_{m}V$, fields from these charges can be calculated using Coulomb’s law:

(5) $${\vec{H}}_{disk}\left(\vec{r}\right)=\frac{1}{4\pi }\sum_{\begin{array}{c} all\\ cells \end{array}}\frac{q_{i}{\vec{r_{i}}}^{\prime }}{{\left|{\vec{r_{i}}}^{\prime }\right|}^{3}}$$

where $q_{i}$ is the calculated bound magnetic charge at the $i^{th}$ cell.

Because these two methods for calculating fields involve summing over all cells each time the field at a particular point is to be calculated, each field calculation requires a large computational time, which is unwanted for the purpose of, for example, dynamic simulations meant to simulate particle motion [40]. To address this, we use a fit that can calculate fields more quickly. To do this, we calculate the magnetic scalar potential $\psi_{m}$ [41], valid as we have no free currents, from which magnetic fields can be obtained using $\vec{H}=-\vec{\nabla }\psi_{m}$. We calculate $\psi_{m}$ using the following formula.

(6) $$\psi_{m}=\frac{1}{4\pi }\sum_{\begin{array}{c} all\\ cells \end{array}}\frac{q_{i}}{\left|{\vec{r}}^{\prime }\right|}$$

We calculate the potential at 276 points (at a height z above the disk) from a point directly above the disk center to a distance of 27.5 µm (or 11 disk radii away). The spacing between points is 100 nm. To these points, we fit the following function:

(7) $$\psi_{1D\;fit}(x,z)=\frac{ax}{dx^{3}+cx^{2}+bx+1}$$

such that the sum of least squares of the difference was minimized and parameters a, b, c, and d are calculated. This fit function was chosen to ensure that the potential was zero in the middle (at x=0) and to have the potential function tend like 1/(distance squared) at distances far from the disk, consistent with the behavior of potential from a dipole. As this function could not perfectly fit the calculated potential, a piecewise function was employed, where in all intervals the same function $\psi_{1D\;fit}\left(x,z\right)$ above was used but with different parameters a, b, c, and d. In our case, for a 5 µm diameter disk, the intervals were 0 to 1.875 µm, 1.875 to 5 µm, 5 to 12.5 µm, 12.5 to 20 µm, and 20 to 27.5 µm. 

From this fit potential function $\psi_{1D\;fit}(x)$, representing a fit to the potential along the x-axis, we calculate the potential at any location in space $\psi_{fit}(\vec{r})$ by assuming that the magnetic scalar potential varies with the cosine of the azimuthal angle (defined as y/x)—in other words,

(8) $$\psi_{fit}\left(\vec{r}\right)=\psi_{fit}\left(x,y,z\right)=\left[\psi_{1D\;fit}\left(x,z\right)\right]\cos\theta =[\psi_{1D\;fit}\left(x,z\right)]\left(\frac{y}{x}\right)$$

**Figure 4:**
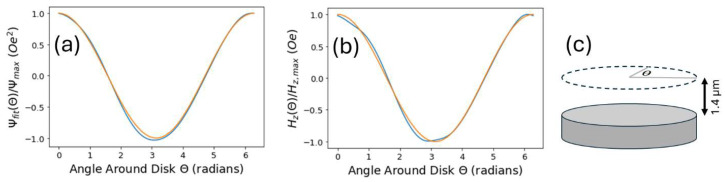
(**a**) Magnetic scalar potential (blue) and (**b**) z-component of disk magnetic field (blue) calculated around the periphery of a disk, 1.4 µm above the disk. (Both curves are calculated using the charge density method). (**c**) Relevant locations where potential and field are calculated in (**a**,**b**) are indicated by the dashed circle, and angle θ is defined. Both the magnetic scalar potential and field vary closely with a cos⁡θ function, illustrated by orange curves in (**a**,**b**).

It is worth noting that, unlike a 100% magnetized disk, the disk properties do not vary perfectly with the cosine of the azimuthal angle. However, in Figure 4 below, we show that both (a) the magnetic scalar potential and (b) the z-component of the magnetic field, both calculated using the charge density method without using a fit, vary with angle much like a cosine curve, suggesting that fitting calculated potentials to a cosine curve is a reasonable approach. 

## 3. Results

### 3.1. Field Calculation Results

**Figure 5:**
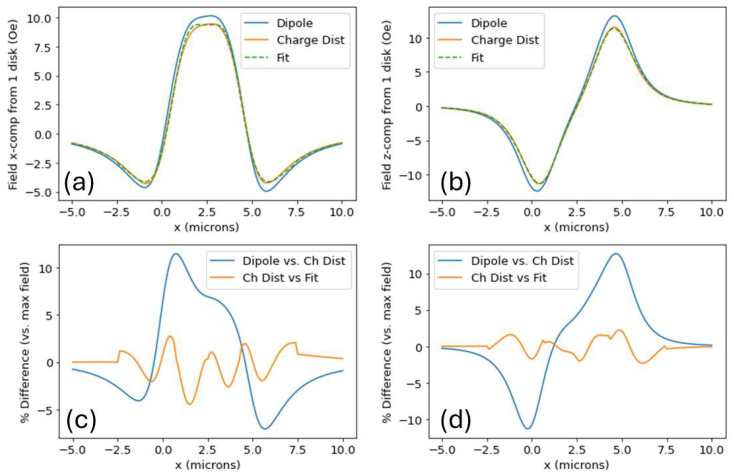
(**a**) ${\vec{H}}_{disk,x}$ vs. x across center of disk for dipole, charge density, and charge density fit calculation methods showing the results are similar. (**b**) ${\vec{H}}_{disk,z}$ vs. x across center of disk and comparison of the three calculation methods. (**c**,**d**) Percent differences (compared to max value) comparing dipole vs. charge distribution and charge distribution and fit methods. Fields are calculated at locations defined by the number line in Figure 1a.

In Figure 5, we show the calculated field from the disk with magnetization shown in Figure 2a, where the fields shown represent the x- and z-components of the field a height of 1.4 um above the disk, along a line parallel to the diameter. The dipole and charge density methods yield fields that differ by <12% (compared to the maximum field in the graph). We suspect the field differences that occur are a result of pixelation effects—the OOMMF models a disk as broken into 40 nm cube cells. Note that the fields shown are the field contributions from the disks themselves and do not include the externally applied fields that magnetize the disk.

Interestingly, the maximum field magnitudes illustrated are only about 12 Oe, yet these fields (and the resulting field gradients) are strong enough to modify a magnetic field landscape such that magnetic microparticles in fluids can be trapped and transported [40]. 

It is worth noting that the charge distribution method is a bit computationally faster than the dipole method, and the fit method is much faster than the other two. To illustrate this, using a desktop computer with 16.0 GB of RAM and a 3.20 GHz processor, it took 4.53 s to produce the dipole field graph in Figure 5b—121 field calculations in all—for an average field calculation of 37.4 ms. For comparison, the charge distribution curve took 2.28 s (18.8 ms/field calculation) and the fit graph took 4.5 ms (37 µs/field calculation). Calculations necessary for Figure 5a required comparable computation times. All computations were performed using Python 3.8.10.

### 3.2. Variation in Stray Fields across Simulations

Field strengths vary dramatically for different disk magnetization landscapes, and OOMMF simulations can yield different results for different simulation runs. For comparison, we discuss three different magnetization configurations: (a) a disk initially randomly magnetized and allowed to relax to an applied 35 Oe in-plane field/50 Oe out-of-plane field, followed by a 360-degree counter-clockwise rotation of the field (in 90-degree increments), resulting in a “vortex” configuration, (b) a disk initially randomly magnetized and then allowed to relax when experiencing an applied 35 Oe in-plane field/50 Oe out-of-plane field, but without the subsequent rotation, denoted “wavy” because of the wavy resulting magnetization landscape, and (c) a fully magnetized disk—denoted “full mag”—with all cell magnetizations pointing horizontally, reminiscent of a disk magnetized by an arbitrarily large in-plane field. (Note that case (a), the vortex, was already used to produce Figure 1, Figure 2, Figure 3, Figure 4 and Figure 5).

While the fully magnetized case is not physically realistic for the small ($H_{ext,x}$ = 35 Oe and $H_{ext,z}$ = 50 Oe) fields employed for the simulation, it was incorporated for the purpose of comparison: a fully magnetized disk would yield the highest possible stray fields. 

**Figure 6:**
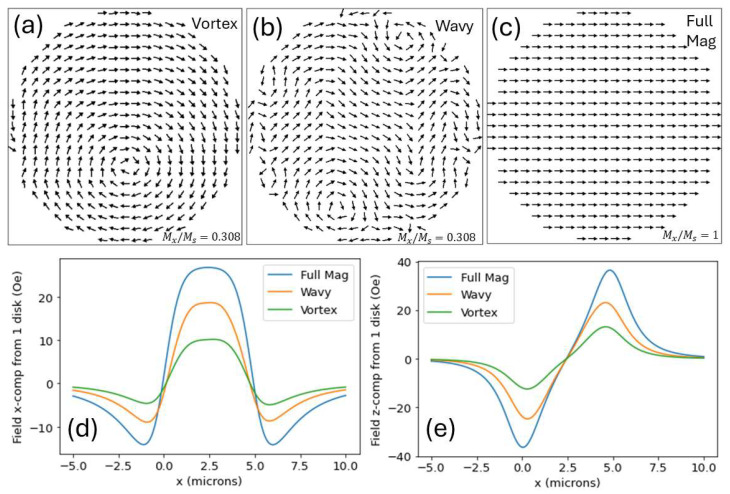
(**a**–**c**) Magnetization landscapes, simulated using OOMMF, used to calculate stray magnetic fields from a disk. (**a**) Vortex, (**b**) wavy, and (**c**) fully magnetized patterns were employed. (**d**) Stray field x-components versus distance along a horizontal axis, 1.4 um above the disk, for each of these landscapes, calculated using the dipole method. (**e**) Stray field z-components calculated along the same horizontal axis, again using the dipole method. (Note that y-components are not included as they were small—less than 3 Oe in all cases).

Our in silico results show that the disk stray fields varied to a large extent even for configurations (a) and (b). This was the case even though the final external field employed by the OOMMF simulation was the same—different initial conditions but identical final conditions yielded maximum fields that differed by almost a factor of 2. (The maximum $H_{disk,z}$ in Figure 6e was 24.8 Oe for the wavy case and 13.2 Oe for the vortex case.) The result of the field rotation producing Figure 6a was the removal of energetically unfavorable domain walls and other locations where the magnetization had large divergences, resulting in a vortex void of all frustration points except the single vortex center. It is important to note that for magnetic particle transport on disks [40], fields are rotated as part of the transport process, so a configuration like 6a is a more likely configuration than what is shown in Figure 6b. Verifying this field calculation model would be possible by measuring fluid-borne magnetic particle responses—particle speed measurements, for example—upon application of trapping forces and manipulation forces from disks.

**Figure 7:**
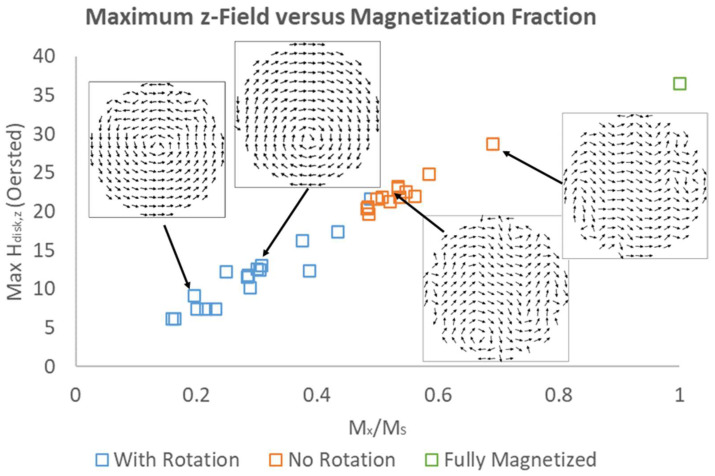
Maximum calculated stray field z-component versus Magnetization Fraction $M_{x}/M_{S}$ for multiple OOMMF simulations produced using methods described in the text. All blue (“With Rotation”) and orange (“No Rotation”) data correspond to a final (simulated) externally applied magnetic field with x and z components of $H_{ext,x}$ = 35 Oe and $H_{ext,z}$ = 50 Oe. A data point for the maximum field from a fully magnetized disk is included for comparison (in green). For several data points, the corresponding magnetization landscape images are included as insets.

It is important to note that upon randomly initializing OOMMF simulations, different magnetization landscapes emerged even for identical final experimental magnetic field conditions. To characterize disk magnetization, we recorded “$M_{x}/M_{S}$”, a metric for how magnetized the resulting disk described by the OOMMF is. (In reality, “$M_{x}/M_{S}$” is the *average* of all $M_{x}$ values in a magnetization landscape divided by the saturation magnetization $M_{S}$). As an example, $M_{x}/M_{S}=$ 0.308 for Figure 6a, 0.533 for Figure 6b, and 1 for Figure 6c (by definition). Stray field maxima increase with $M_{x}/M_{S}$, as seen in Figure 7. It is worth noting the variation in simulation results even when the setup of the simulation was identical. All blue data points represent identical OOMMF simulations (except for the random magnetization initialization), and likewise for orange data points. Despite this, the standard deviation of $M_{x}/M_{S}$ values for “identical” simulations was rather large. For 10–15 simulations, $M_{x}/M_{S}$ = 0.286 ± 0.092 for the simulations with rotation and 0.536 ± 0.056 for simulations without rotation. The magnetization landscapes in plots 6(a) and (b) were chosen as representative cases near these averages.

## 4. Discussion

To summarize, we explore methods for calculating magnetic fields in the vicinity of permalloy disks by using micromagnetic simulations. Regardless of whether fields were calculated directly from dipoles within the material, from calculated bound charge distributions, or from the magnetic scalar potential fit method described, reasonably similar field calculations emerge. However, for the weak applied magnetic fields investigated here and used experimentally to trap and transport magnetic microparticles [40], small or seemingly insignificant differences in OOMMF simulations can yield drastic changes in resulting calculated disk magnetization landscapes and stray fields. The micromagnetic simulations studied suggest that more than just a final external field configuration is necessary for obtaining a reliable understanding of the micromagnetic properties of a disk—the steps taken to get to that result influence the result as well.

## Data Availability

The data from this article are available by request.
